# Supplementary material for: The Systems Biology Research Tool: evolvable open-source software
Source: BMC Syst Biol. 2008 Jun 29;2:55. doi: 10.1186/1752-0509-2-55 (PMC2446383; doi:10.1186/1752-0509-2-55)
Supplement: Additional file 1 — SBRT Archive. An archive of the current version of the Systems Biology Research Tool. [file 1752-0509-2-55-S1.zip › sbrt-1.4.0/doc/developers_guide/api/sbrt/kernel/math/package-frame.html]

sbrt.kernel.math


sbrt.kernel.math

Interfaces 
  
*EquivTester*
  
*LinearComb*
  
*LinearComb.Term*
  
*MathExpr*
  
*Polynomial*
  
*Polynomial.Term*
  
*QuadraticExpr*
  
*QuadraticExpr.Term*

Classes 
  
AbstractLinearComb
  
AbstractLinearComb.Term
  
AbstractPolynomial
  
DoubleEquiv
  
HashLinearComb
  
HashLinearComb.Term
  
Interval
  
IntervalComparer
  
IntervalComparer.Diff
  
IntervalEquiv
  
VectorComparer
  
VectorComparer.Diff

Exceptions 
  
ConstraintViolationException
  
ExceededLimitException
  
IllegalBoundsException
  
InfeasibleSolutionException
  
InvalidPointException
  
MissingVariableException
  
UnboundedVariableException
